# In-depth interviews with state public health practitioners on the United States National Physical Activity Plan

**DOI:** 10.1186/1479-5868-10-72

**Published:** 2013-06-04

**Authors:** Kelly R Evenson, Sara B Satinsky, Cheryl Valko, Jeanette Gustat, Isobel Healy, Jill S Litt, Steven P Hooker, Hannah L Reed, Nancy O’Hara Tompkins

**Affiliations:** 1 Department of Epidemiology, Gillings School of Global Public Health, University of North Carolina Chapel Hill, 137 East Franklin Street, Suite 306, Chapel Hill, NC 27514, USA; 2 Prevention Research Center, Brown School of Social Work, Washington University in St. Louis, St. Louis, MO 63110, USA; 3 Department of Epidemiology, Prevention Research Center, School of Public Health and Tropical Medicine, Tulane University, New Orleans, LA 70112, USA; 4 Department of Environmental Health, Colorado School of Public Health, Aurora Colorado 80045, USA; 5 Exercise and Wellness Program, School of Nutrition and Health Promotion, Arizona State University, Phoenix, AZ 85004, USA; 6 WV Prevention Research Center, Department of Social and Behavioral Sciences, School of Public Health, West Virginia University, Morgantown, Morgantown, WV 26506, USA

**Keywords:** Evaluation, Intervention, National plan, Physical activity, Populations, Surveillance

## Abstract

**Background:**

The United States National Physical Activity Plan (NPAP; 2010), the country’s first national plan for physical activity, provides strategies to increase population-level physical activity to complement the 2008 physical activity guidelines. This study examined state public health practitioner awareness, dissemination, use, challenges, and recommendations for the NPAP.

**Methods:**

In 2011–2012, we interviewed 27 state practitioners from 25 states. Interviews were recorded and transcribed verbatim. Transcripts were coded using a standard protocol, verified and reconciled by an independent coder, and input into qualitative software to facilitate development of common themes.

**Results:**

NPAP awareness was high among state practitioners; dissemination to local constituents varied. Development of state-level strategies and goals was the most frequently reported use of the NPAP. Some respondents noted the usefulness of the NPAP for coalitions and local practitioners. Challenges to the plan included implementation cost, complexity, and consistency with other policies. The most frequent recommendation made was to directly link examples of implementation activities to the plan.

**Conclusions:**

These results provide early evidence of NPAP dissemination and use, along with challenges encountered and suggestions for future iterations. Public health is one of eight sectors in the NPAP. Further efforts are needed to understand uptake and use by other sectors, as well as to monitor long-term relevance, progress, and collaboration across sectors.

## Background

A high prevalence of physical inactivity and resulting harmful health effects, coupled with evidence for effective promotion strategies [1] make physical activity a global public health priority [2]. The 2010 Toronto Charter for Physical Activity describes several worldwide calls for action, including implementing a national policy and creating country-level plans to foster population increases in physical activity [3]. Country-level plans typically include a series of policy and/or practice recommendations intended to increase population-level physical activity, goals for the country’s physical activity, details of how the plan was created, and data to support recommendations [4-6]. The United States (US) National Physical Activity Plan (NPAP) is the first national-level plan in this country to focus exclusively on physical activity [6] and follows the physical activity guidelines released in 2008 by the US Department of Health and Human Services [7].

The NPAP outlines population-based strategies to increase physical activity across eight sectors [6]. The sectors, each with white papers summarizing the evidence base, include business and industry [8]; education [9]; health care [10]; mass media [11]; parks, recreation, fitness, and sports [12]; public health [13]; transportation, land use, and community design [14]; and volunteer and non-profit organizations [15]. The NPAP includes five overarching strategies and 44 sector-specific strategies, with corresponding tactics to address them. A mix of organizational partners and experts serve on the NPAP Coordinating Committee to guide plan efforts [16]. The NPAP was launched to a national audience in 2010. Media outlets promoted the plan launch and organizational partners were encouraged to champion the NPAP through their respective communication channels. Some partners created newsletters or press releases, while others held scientific sessions at national meetings. A few months after the 2010 launch, a group led by the National Coalition for Promoting Physical Activity released an implementation plan focused on the sectors to support initial NPAP efforts [17]. Upon release of the implementation plan, partners in six of eight sectors organized and began meeting regularly to work on goals [18].

Guidance is lacking on best practices for dissemination of evidence-based physical activity interventions, particularly with a focus on policy. Moreover, dissemination of national plans is often not evaluated [4]. Therefore, we conducted in-depth interviews with state-based public health practitioners to determine early awareness, dissemination, uses, challenges, and future recommendations of the NPAP and the companion implementation plan. These findings have implications for other national physical activity plans, as well as plans addressing other chronic disease risk factors.

## Methods

### Sample

Interviews were conducted with either the lead state physical activity practitioner, as designated by the Centers for Disease Control and Prevention (CDC), or the person at the state health department most knowledgeable about the organization’s physical activity promotions and programs. To obtain geographic representation, we emailed and telephoned people in a convenience sample of states from each of four US census regions (defined elsewhere: http://www.census.gov/geo/www/us_regdiv.pdf). Interviews were conducted between July 2011 and March 2012. While we did not select states by whether or not they had a state wide plan that included physical activity, at the time of the interviews all states in the sample had a state wide plan with a focus on physical activity, usually within an obesity or chronic disease prevention plan.

### Interview guide

The Institutional Review Boards at each collaborating site approved our standardized protocols. We developed an interview protocol, guided by the RE-AIM (Reach, Effectiveness, Adoption, Implementation, Maintenance) framework [19] and Diffusion of Innovations theory [20]. This guide included questions to assess awareness, dissemination, uses, challenges, and future recommendations of the NPAP (Appendix). The guide also included items about awareness and use of the implementation plan, and descriptive questions on the interviewee’s job title and verification of their state plan that incorporated physical activity.

Twenty-three interviews were conducted over the telephone and two were conducted in person. Two telephone calls included two people interviewed simultaneously. A fact sheet and copy of the interview questions were sent beforehand. Verbal consent was obtained at the start of the interview. All but one interview was recorded and transcribed verbatim. One participant declined to be recorded; therefore, detailed notes were taken.

### Analysis

The codebook, initially developed from the interview guide, was expanded with new codes that emerged during the coding process. It consisted mainly of deductive codes generated from the research questions and interview guide. Additionally, both uses and challenges were coded using characteristics that tend to promote higher diffusion, including compatibility, evidence-based, complexity, flexibility, risk, trialability, reversibility, observability, implementation, cost, and relative advantage [20]. As part of central training, all collaborators coded one transcript and compared their codes against a guide created by the study lead. Discussions were held to clarify issues and achieve consensus. Following training, all transcripts were then double coded with a central person serving as the second coder. Any discrepancies in coding were resolved by consensus. Coded transcripts were then entered into ATLAS.ti (version 5.2.17) to facilitate generation of themes. At least two people analyzed the resulting output of quotes accompanying each code. The transcriptions for each interview were then reviewed by a single person to verify themes and for other pertinent information not deduced from coding.

## Results

### Description of sample

Twenty-seven state public health practitioners were interviewed, representing 25 states in each of four US regions (6/12 Midwest, 5/9 Northeast, 8/16 South, 6/13 West). Among the sample, 19/27 were members of the National Society of Physical Activity Practitioners in Public Health (NSPAPPH), a professional organization of physical activity practitioners in public health (http://www.nspapph.org/).

### Awareness and dissemination of the NPAP

All but two respondents interviewed were aware of the NPAP. One non-aware respondent stated, “I know about the physical activity guidelines, and I wonder if they were one and the same.” The other respondent not aware of the NPAP stated, “I don’t know that plan. So I haven’t really read up on it to share it. All we do is we come up with our own plan and we share it.” Most respondents first learned of the plan before the initial launch in 2010, and several learned of it after the launch. Some did not recall when they learned of it, as exemplified by one respondent: “I’m not sure exactly when because I get several plans across my desk pretty much monthly.”

Participants primarily learned of the NPAP through communications from the NSPAPPH, the CDC, and at national and state meetings. Several interviewees also helped with the plan creation, while others learned of it when hired into their position. Most respondents shared the NPAP with others, often involving further discussion such as through webinars or meetings. For example, one interviewee “did a state wide seminar when it first came out” and another stated that “the first thing I did after the plan was rolled out was I brought together … seven different departments here at the state … and I educated them on the plan and just basically did a state rollout here. And then we also sent this similar information and held a teleconference call for the counties.” Some of the state practitioners shared the NPAP with local practitioners: “We’ve put it out in our email listserv to local public health and others interested in physical activity.” However, others did not share it: “I personally haven’t sent it or shared it with any of our local [practitioners].” Only a few of the respondents were currently involved with the NPAP sector activities. For example, one respondent stated that “it was not clear that physical activity practitioners could participate.”

Related to awareness, respondents were asked if the leadership and staff at their organization were aware of the NPAP. Most indicated staff were familiar with the NPAP, but split approximately equally on leadership awareness. “They haven’t explicitly encouraged use of the plan; however, in my role I’m sort of responsible to identify what resources are available, what plans are available that we can coordinate with and implement, so I think that it’s sort of implied from leadership that we take advantage of the best resources that are available. And I think the National Physical Activity Plan is something that we’ve selected to sort of work from in our work. So if that makes sense, I don’t think it’s been like a directive to use it, but we have used it because we see the value in it.” Several respondents attributed lacking support for the NPAP among their leadership due to other competing priorities and plans, including their own state plan. As one respondent remarked, “There’s an awful lot of plans out there”.

### Positive uses and challenges of the NPAP

Respondents were asked to describe both positive uses and challenges of using the NPAP, grouped into themes. The most common positive use was as a reference document to develop state-level strategies and goals. For example, “we're using some of the ideas in the National Plan to develop our own plan” and “it provided the references and the resources and the tools that we needed to shape that [state obesity] plan.” Most state practitioners were not using the NPAP to implement strategies, and instead referred to their state-based plans that included physical activity. Other respondents noted the usefulness of the NPAP as a guidance tool for coalition building and a resource tool for local practitioners: “I really use that a lot for helping to form coalitions, using it as guidance, and more using the plan as a resource for our local partners.” Another respondent noted is usefulness in grant applications: “We have a grant program here … and in this particular grant program all the grantee agencies are required to have a physical activity component and they are advised to use the plan to direct their physical activity component.”

The NPAP supported existing work for some respondents. For example: “it validated what we’ve been doing all these years”; “in a way nice to see that the work that we were doing when the plan came out was consistent with what the plan was saying”; and “this allowed me to reach out to our other sister agencies to kind of just justify the work we’ve been doing and to help us move forward a little more systematically and just engage them at a higher level.” A few respondents said that after the NPAP was released they made immediate revisions to their state plan. “We did a significant edit of our objectives. And we specifically looked at … the objectives in the physical activity plan to make sure that we were capturing everything and so that it is consistent.” Others also used the NPAP when revising their state plans. For example, “We updated our state’s obesity prevention plan last year, and we did incorporate a lot of the strategies.” However, most state-level practitioners could not provide examples of the NPAP being used at the local level.

The NPAP supported environmental and policy changes. One respondent stated that “when the new physical activity guidelines came out in 2008, I think it was sort of like the elephant in the room. Here are these guidelines, we know we need to get more physical activity, but the environment isn’t conducive to that, or there [are] no sidewalks in my neighborhood, that sort of thing. So the National Physical Activity Plan really addresses that and what we can do to improve the environment and the policies that support the environment to get more physical activity.”

Several themes emerged around challenges, particularly insufficient time and resources to implement the NPAP. “I think as it stands right now the economic message is probably bigger than any other message. How can we do this on the cheap?” Three themes relating to challenges were balanced by positive aspects, with exemplary quotes provided in Table 1. First, most respondents identified the NPAP as useful in making physical activity a priority or in using ideas from the NPAP to develop their own state plan. In contrast, others felt the strategies in the plan needed further development to be useful, particularly around implementation. Second, some respondents described the NPAP as being concise, while others said it provided a broad overview but did not help with implementation, making it more difficult to use. Other respondents explained that, with the large number of tactics outlined, it was hard to know where to begin and what to prioritize. Related to this, some respondents mentioned other competing reference documents pertaining to physical activity, and were confused as to how they fit together with the NPAP. Third, some respondents noted the NPAP was compatible with their existing state plan or goals, but others said it lacked compatibility with federal policy recommendations.

**Table 1:** Quotes that exemplify positive uses and challenges

**Positive uses**	**Exemplary quotes**	**Challenges**	**Exemplary quotes**
Usefulness	“… there wasn’t a whole lot of focus put on the physical activity components of some of the grants. It was more like nutrition, nutrition, nutrition. And now we’re adding physical activity right up there as an equal partner. So I think that probably helped a lot.”	Not as useful	“We can talk about the strategies that are outlined in the plan, but finding kind of those meeting points between health and community design sometimes are very difficult.”
	“…it gave us more of a focus area for things that we would want our grantees to focus on…”		“I still wonder how we all want to see this really being effective and who is it for? And I think those are huge questions, like who is this really for? If it’s for the general public, then we really need to nail it down. If it’s really kind of a document for people like you and I that do this work, then I think the format that it is, is going to work fine.”
	“the strategy language and the goal language and so forth. That's been very helpful”		
	“…we used [the NPAP] to help devise our plan as far as what we were going to do for programming at the state level and grants to the local level.”		
Simplicity	“I think that it's a fairly concise document for the amount of information that it includes, but … and so for someone who doesn't have a lot of background in physical activity, that it kind of provides them a quick, broad, general picture of the efforts that can be done regarding physical activity.”	Complexity	“…while it provides a very broad overview, it doesn't actually provide you with how you can implement these type of strategies and how you can actually integrate it into your work.”
			“Having a simplified [plan] where you can click on if you’re a worksite or if you’re a school or even another category, we use what we call, we do healthy in front of all of our website categories, healthy schools, healthy childcare, and then we do healthy you, so there’s also a piece that would even wrap it in the [physical activity] guidelines. But I think we need to keep the language as simplified as possible because I think we’ve just gone all over the map.”
Compatible or consistent	“We can move forward with what we did and still be in line with the National Plan.”	Not compatible or consistent	“There seems to be a disconnect with maybe some of the federal policy recommendations and the Physical Activity Plan…. I think that the other federal organizations could do a better job of supporting that at the national level.”
	“Most of the objectives of the National Plan actually almost mirrored or complemented our state plan.”		

### Future Iterations of the NPAP

Respondents were asked to provide suggestions to improve future iterations of the NPAP (Table 2). The most common suggestions pertained to implementation materials, in particular to link strategies and tactics to specific examples, resources, and lessons learned. One respondent summarized this idea stating that the NPAP “broke it into settings, which was helpful, into strategies, which was helpful, and then into tactics within that, but then that’s sort of where it stopped.” Another participant remarked that “the strategies and tactics were listed, but then the follow-up on what was suggested or recommended, if that exists, I don’t know where it exists, because I haven’t seen them.” Of relevance, participants suggested enhancing the website (http://www.physicalactivityplan.org/) by connecting strategies and tactics in the plan to exemplary programs.

**Table 2:** Suggestions for future iterations of the NPAP

**Suggestion for improvement**	**Exemplary quotes**
Link the NPAP tactics to examples, resources, and lessons learned	“I don't know if there are specific programs linked anywhere, like on their website…. If there was some way to connect with programs that already exist so states could replicate, that would be helpful.”
	“I do think the messages need to be simplified with some great local examples and then a couple of resources where people can go.”
	“I think examples from other states that have used the plan in various sectors and how they’ve been able to … like the different strategies and tactics used and the lessons learned.”
	“…if they have any implementation pieces, but there’s some specific story examples. Those tend to be the most powerful things for me… those stories of people that have had success are key I think.”
	“I guess maybe we’ve been living in these strategies for quite a while, it’s almost to the point now where we want to have some direct examples we can give people for implementation.”
	“I think part of it is it's difficult to find that there are really tools in it to help with planning or implementing the tactics that they outline… [such as] resources that helped you plan or implement the tactics that are outlined.”
Provide shorter synopsis documents of the plan	“I’d like to see … a synopsis… Because whenever I go to places and provide information on the plan, of course I’m not going to bring it with me, it’s so huge, and I give the website so people can download it if they’d like, but I’d like to have like a little synopsis of the plan that would be like maybe a one page, maybe two if you turned it over, that would give a nice little synopsis of it, something that we could take around with us whenever we give information on the plan would be very helpful.”
Tools to communicate with partners outside of public health	“…is how to talk about the National Physical Activity Plan to outside partners. So let’s say if I’m talking to the Department of Transportation to see what are some tools that I can get to kind of talk about the plan and talk about how Public Health intersects with Transportation and how to use the Physical Activity Plan. Or if I’m talking to the Department of Recreation, how to use this too, how to talk about the Plan. So just basically some tools to be able to communicate with other sectors on how we can use this plan for mutual benefit.”
Provide sample work plans using SMART objectives that use the NPAP strategies and tactics	“…if there were a sample like work plan section … that would have some examples of how to use the objectives in the plan and to have them written in like a SMART objective as an example that could be used for work plans for grants for example, that sort of thing. Or ideas on how to use the strategy in real life events.”
Webinars	“You know, webinars are always really useful, I think. I’m very knowledgeable about what the plan contains, but when you try to explain that to our local health department personnel, a lot of them don’t have the background in public health.”
Encourage other organizations to use the plan	“…put in there that part of the requirement is that … you need to incorporate some of the National Physical Activity Plan language in that grant application will help make that stronger, or they’re going to have to look at it as part of their guidance, or use it as a guidance.”
	“I think it would be helpful if the national organizations refer to it more often. I think that would increase the credibility of it.”
Marketing	“I think there needs to be a better marketing plan somehow.”
Evaluation	“I think the best thing that the National Plan could do would be to come up with some measures, some indicators and some evaluation measurements that they feel should be measured across the United States. And so that we could all be measuring similar things so that we can actually get a picture of what’s going on, because if we each have different indicators and different evaluations, measurements, I don’t think we’re going to be able to compare across states.”
	“I’m sure it [the NPAP] talks about evaluation somewhere, but I don’t see that it really gives you any information on where to find that.”
Identify the costs of tactics	“So this is a low cost intervention, this is the medium cost, this is the higher level cost. So then you have two pieces. You have the people that have actually done it, the stories coming from the field. And then … if it’s a municipal or a school, they want to see low cost. They don’t have the money to put into it. So if you kind of highlighted both of those things, successful things that don’t cost a lot of money, I think that would be a lot of bang for the buck.”

Other more frequent suggestions were to create shorter synopsis documents for each sector, and tools to communicate with partners outside of public health. One respondent suggested providing work plan examples that correspond to plan tactics with SMART criteria (Specific, Measurable, Achievable, Realistic, Timely) [21] for future goal setting. Other ideas included creating webinars, particularly to reach local health departments, and marketing materials. Several respondents mentioned the NPAP should be incorporated into grant applications, and proposed that other organizations use the plan to increase its credibility. Several respondents suggested identifying the general costs for various tactics; for example, categorizing them into low, medium, and high cost interventions. Less frequent suggestions included evaluation guidance for tactics.

### Implementation plan

Approximately half of the state-level practitioners were aware of the companion implementation plan titled “Make the Move: 2010–11 National Implementation of the US Physical Activity Plan” [17]. One respondent said that the implementation plan was easier to use “because it’s more like what we’re doing. I guess maybe we’ve been living in these strategies for quite a while, it’s almost to the point now where we want to have some direct examples we can give people for implementation.” Three practitioners informed us that they have used or plan to use the implementation plan as a guidance document for strategy development. However, most practitioners aware of the implementation plan said they rarely or never used it to inform their work and several reported they preferred to refer directly to the NPAP. Due to infrequent use, challenges to utilizing the implementation plan were not often mentioned. However, one respondent noted a lack of focus on rural communities. “We may not be doing all of it because we’re really a rural state, so sometimes things are a little different here …so having a few more examples of how things could work in really rural states [would help]”. Another participant mentioned it would be helpful if recent findings from implementation strategies were posted on the NPAP website.

## Discussion

Interviews with state public health practitioners provided insights into the initial awareness, dissemination, uses, challenges, and future recommendations for the NPAP since its launch in 2010. Applying the RE-AIM framework [19], we found high awareness (reach) of the NPAP among the state-level practitioners we interviewed. The channels through which they learned of the plan varied. This implies that regular communication through several different communication channels (e.g., website, newsletters, webinars, listservs) would continue to inform and engage practitioners. This would strengthen the NPAP’s relevance, remind practitioners of its intent and usefulness, and may be particularly important considering staff turnover within public health agencies [22,23].

Dissemination of the NPAP through state practitioners to local health departments and coalitions was inconsistent. Among those who disseminated the NPAP locally, most did so only one time. Yet, the interviewees had high awareness of the plan and served as state wide contacts for local practitioners, making state practitioners a possible conduit to communicate with local practitioners. Given that there are 59 state and territorial health departments and more than 3000 local health departments in the US [24], state-level practitioners could have substantial reach, beyond current levels. Such dissemination efforts could be augmented by providing state-level practitioners with example text and summary documents to send local partners. Among the public health workforce, only 44% are identified as health professionals, and even fewer are specifically trained in public health [22]. Thus, assumptions that all practitioners understand how to use the NPAP should not be made. There was also inconsistency in bringing awareness of the NPAP to leadership within state health departments. Further promotion with targeted tools and materials related to the NPAP may enhance this targeted outreach.

Most respondents either were not aware of the companion implementation plan or had rarely or never used it. This lack of use was evident in suggestions to connect the NPAP’s strategies and tactics directly to implementation examples. This finding demonstrates a significant opportunity to enhance NPAP implementation. It may be useful to bundle the documents and resources together, to facilitate connecting a strategy or tactic to implementation guidance. More broadly, there was confusion by a few respondents about overlap in several major physical activity documents, including the NPAP and its implementation plan, the 2008 Physical Activity Guidelines for Americans [7], the Guide to Community Preventive Services (http://www.thecommunityguide.org) [1], and the Community Tool Box (http://www.ctb.ku.edu/en/default.aspx). Development of a national-level document that summarizes the critical reports, guidelines, and tools pertaining to physical activity, and electronically linking to each of them for easy access, may help facilitate a better understanding of the guidance and resources available and how best to use and distinguish among them from one another.

A 2010 survey of NSPAPPH members corroborated several findings from these interviews [25]. The survey indicated high awareness of the NPAP among NSPAPPH members, more so among state than local practitioners, and low awareness of the implementation plan. Few survey respondents agreed that the NPAP was effectively disseminated to physical activity practitioners in their state. Only about half of those respondents reported awareness of the NPAP among leadership and intervention staff at their workplace. Limited resources were identified as a barrier to implementing the NPAP, similar to what emerged from our in-depth interviews.

The Diffusion of Innovations theory suggests that specific attributes may contribute to the speed or extent that an innovation is disseminated [20,26,27]. Applying this to the NPAP, and without prompting by the interviewers on positive aspects and challenges to using the NPAP, the plan was described by some respondents as useful, simple, flexible, and compatible with existing state plans. Each of these attributes helps facilitate the dissemination of the NPAP. However, we also identified ways to make the plan more useful, particularly for implementation, noting that several respondents described the plan as complex or incompatible with federal policies. Moving forward, a focus on addressing these characteristics as suggested in recommendations below may widen the use and adoption of the plan. Moreover, the forthcoming book on specific case examples of best practices can aid those intending to use the NPAP [28].

The most frequent positive use of the NPAP was as a reference to development of state-level goals. To enhance use of the NPAP instead as a direct guide for state-level planning, new materials could describe how to use the national plan, particularly for states updating their health-related plans or developing stand-alone physical activity plans [29]. Other research indicates that challenges to implementing evidence-based public health strategies include organizational factors, such as staff turnover, limited resources, lack of policy maker’s support, lack of rewards for using evidence-based practices, and individual factors, such as lack of time, knowledge, and communication skills [24,30,31]. Addressing these factors can also enhance dissemination of the NPAP.

A typology for using research evidence in practice can be applied to also help illustrate how the NPAP is being used [32]. *Instrumental use* occurs when research evidence is directly applied to decision-making, such as using the NPAP to assist with development of state plans. *Conceptual use* refers to situations in which research evidence influences how practitioners think about issues, problems, or potential solutions. For the NPAP, several respondents mentioned that learning from non-traditional sectors (transportation) influenced their thinking. *Tactical*, *political*, *or symbolic use* occurs when research evidence is used to justify particular positions, such as the NPAP validating their own plans or work. The final use, *imposed use*, is defined as situations in which there are mandates to use research evidence, such as when government funding requires practitioners to choose strategies from the NPAP. The authors note that the typology is not exhaustive, nor mutually exclusive, but demonstrates how research evidence (in our case the NPAP) serves multiple purposes and is used in multiple ways.

In the US, this is the first national-level plan to focus exclusively on physical activity, though national plans exist for a number of related health behaviors and diseases, such as tobacco control [33], diabetes mellitus [34], cancer [35], and cardiovascular disease [36,37]. Building upon the suggestions of practitioners, we offer several recommendations. These suggestions have implications for other national plans and include:

Providing short synopses of different sections to assist users in digesting information, particularly for those only interested in one sector in the case of the NPAP.

Using diverse communication channels to distribute the plan and to provide regular plan updates to maintain awareness and increase use of the plan.

Linking implementation strategies and examples to the plan.

Grouping strategies by cost and impact into high, medium, and low categories.

Offering two-way communication (rather than one-way, focusing on streaming the plan to practitioners) to enhance dissemination [26]. For example, a social network could be created through sites such as Community Commons (http://www.communitycommons.org/) where information can be shared and professionals from different sectors with similar interests can connect.

Developing a national-level document that summarizes the critical reports, guidelines, and tools pertaining to physical activity together, and electronically linking to each of them for easy access, to facilitate an understanding of the guidance and resources available and how best to use and distinguish among them.

### Strengths and limitations

We gathered perspectives from state public health practitioners about their awareness and use of the NPAP. The sample included broad geographic coverage, with half of all states represented. We met our goal number of interviews across census regions; however, it is unclear whether respondents were more likely to be familiar with and use the NPAP than those who did not participate. Despite the geographic representation, the absolute sample size limited our ability to explore regional variations. Moreover, awareness of the NPAP may have been over reported, since interview questions were sent to participants prior to the interview.

## Conclusion

There are substantial challenges in translating scientific evidence into meaningful public health programs and policies [38]. Currently, there is little research on best practices for dissemination of evidence-based physical activity interventions [39], especially those targeting policy [40] and underserved populations [41]. We interviewed public health practitioners to assess dissemination of the NPAP, an evidence-based plan to increase population-levels of physical activity. The results provide early evidence of dissemination and use of the NPAP, along with challenges encountered and suggestions for future iterations. It is noteworthy that public health is one of eight sectors in the NPAP. Further efforts are needed to understand uptake and use by other sectors, as well as to monitor long-term relevance, progress, and collaboration across sectors. The lessons learned from this work can be applied to implementation of other national plans.

## Appendix

Interview questions for this study (without probes and verbal scripts)

About Your Job

(1) Please state your job title and describe your role.

(2) In what city and state do you work?

(3) How long have you been in this role?

About You and the National Physical Activity Plan

(4) Have you heard of the National Physical Activity Plan?

(5) In what context did you learn about the Plan?

(6) Have you provided feedback on the Plan to the National Physical Activity Plan Group since it was published in 2010?

(7) Have you participated at the national level with any of the sector committees, either before or after the National Physical Activity Plan was published?

(8) Since learning about the Plan, have you used it in your work?

(9) What parts of the Plan are most useful to you and your work? Have any positive changes occurred?

(10) Are there any difficulties you have had in using the Plan?

(11) Describe in detail the challenges you have had in working with the Plan.

(12) What supplementary materials or activities would make the Plan more useful to you and your work?

(13) Do you have any ideas for how the Plan content or structure could be made more useful to you and your work? For example, what would you like to have in the Plan that is not in it currently?

(14) Have you shared the plan with others in your state?

About Your Organization and the National Physical Activity Plan

(15) Is the leadership at your organization familiar with the Plan?

(16) Are those you identify as the intervention or programmatic staff where you work familiar with the Plan?

(17) Did the Plan change your organization’s activities at the state or local level?

(18) Have staff scheduled periodic assessments of how the Plan is used in organizational planning and activities?

About Your State and Local Uses of the National Physical Activity Plan

(19) Are you aware of the Plan being used at the local level in your state?

(20) Does your state currently have a physical activity related state plan?

Implementation Plan

(21) Are you familiar with the national implementation plan (for the National Physical Activity Plan)? The publication is titled “Make the Move: 2010–2011 National Implementation of the U.S. Physical Activity Plan”.

Other Questions

(22) Can you tell me of other colleagues, and their affiliations, in your state working with the National Physical Activity Plan that we may want to interview?

(23) Do you have any additional comments to share before we end the interview?

## Abbreviations

CDC: Centers for Disease Control and Prevention; NPAP: National Physical Activity Plan; NSPAPPH: National Society of Physical Activity Practitioners in Public Health; RE-AIM: Reach Effectiveness Adoption Implementation Maintenance; SMART: Specific Measurable Achievable Realistic Timely; US: United States

## Competing interests

The authors declare that they have no competing interests.

## Author contributions

KRE oversaw the development of the study, the drafting of the interview guide and procedures, the analysis of the transcripts, and drafted the article. All coauthors assisted with the development of the interview guide, assisted with coding and analyzing the transcripts, reviewed the article several times, and gave final approval for its submission. In addition, SBS, CV, IH, SPH, HLR, and NOT conducted interviews. All authors read and approved the final manuscript.
